# Temporal transcriptome analysis suggest modulation of multiple pathways and gene network involved in cell-cell interaction during early phase of high altitude exposure

**DOI:** 10.1371/journal.pone.0238117

**Published:** 2020-09-10

**Authors:** Priya Gaur, Supriya Saini, Koushik Ray, Kushubakova Nadira Asanbekovna, Almaz Akunov, Abdirashit Maripov, Akpay Sarybaev, Shashi Bala Singh, Bhuvnesh Kumar, Praveen Vats

**Affiliations:** 1 Defence Institute of Physiology and Allied Sciences, Delhi, India; 2 Kyrgyz Indian Mountain Biomedical Research Centre, Bishkek, Kyrgyz Republic, Kyrgyzstan; 3 National Institute of Pharmaceutical Education & Research, Hyderabad, Telangana, India; Universidad de Jaen, SPAIN

## Abstract

High altitude (HA) conditions induce several physiological and molecular changes, prevalent in individuals who are unexposed to this environment. Individuals exposed towards HA hypoxia yields physiological and molecular orchestration to maintain adequate tissue oxygen delivery and supply at altitude. This study aimed to understand the temporal changes at altitude of 4,111m. Physiological parameters and transcriptome study was conducted at high altitude day 3, 7, 14 and 21. We observed changes in differentially expressed gene (DEG) at high altitude time points along with altered BP, HR, SpO_2_, mPAP. Physiological changes and unsupervised learning of DEG’s discloses high altitude day 3 as distinct time point. Gene enrichment analysis of ontologies and pathways indicate cellular dynamics and immune response involvement in early day exposure and later stable response. Major clustering of genes involved in cellular dynamics deployed into broad categories: cell-cell interaction, blood signaling, coagulation system, and cellular process. Our data reveals genes and pathways perturbed for conditions like vascular remodeling, cellular homeostasis. In this study we found the nodal point of the gene interactive network and candidate gene controlling many cellular interactive pathways VIM, CORO1A, CD37, STMN1, RHOC, PDE7B, NELL1, NRP1 and TAGLN and the most significant among them i.e. VIM gene was identified as top hub gene. This study suggests a unique physiological and molecular perturbation likely to play a critical role in high altitude associated pathophysiological condition during early exposure compared to later time points.

## Introduction

Reduced barometric pressure with increased altitude concurrently reduces the inspired oxygen partial pressure in humans [1]. In spite of reduced partial pressure of inhaled oxygen to the cells and tissues body adjusts to survive the extreme hypoxic environment referred as acclimatization [2]. These adjustments results in series of physiological responses to maintain adequate tissue oxygen delivery and supply at high altitude [3]. Increased pulmonary ventilation, an increase in cardiac output by increasing heart rate, changes in vascular tone, as well as increased hemoglobin concentration are well reported and observed as compensatory mechanism [4–6]. These responses to altitude are dependent upon individual to individual, their history of exposure to altitude, ventilation physiology, age, and altitude attained and stay [7, 8]. Individuals un-acclimatized to altitude are bit prone to altitude induced immediate effects like acute mountain sickness (AMS). It is the consequence of severe AMS that can lead to life threatening conditions like high altitude pulmonary edema and high altitude cerebral edema [9]. AMS is not life threatening if occur at initial days of altitude stay and then gradually subsides. There are several physiological, metabolic, and transcriptional level changes observed in humans, which can cause initial AMS and prevalence of AMS signs during preliminary altitude acquaintance [10, 11]. Highland area which had gained interest in past few years is Tien- Shen and Pamir mountain ranges in Kyrgyzstan. Very less is known about the Kyrgyzstan adaptation feature, besides few studies [12, 13]. Kyrgyz acclimatization responses at an altitude of 3200m recently published by us was the first study to explore the signaling by genome wide expression analysis associated with hypoxia exposure [14] but the gene expression at high altitude >4,000 m remains unexplored in Kyrgyz.

We analyzed the time based exposure induced perturbation in physiological and gene expression pattern at high altitude in Kyrgyz. Global transcriptome analysis using RNA sequencing, recent advanced technique, could effectively detect thousands of genes and their consecutive expression patterns. Systemic bioinformatics tool could further enhance the data presented by the RNA sequencing. Time series analysis benefits biology to dynamically capture the complete drift impinges in humans and specific selection of decisive time point for further therapeutic interventions. Present study was done with an objective to comprehend the responses during long 21 days stay at altitude of 4,111m in Kyrgyzstan by comparing the temporal response at each time point as compared to basal response. We studied molecular orchestration by enriched pathway analysis, gene clustering, and network analysis to find out critical time point indicative of acclimatization and top candidate genes controlling acclimatization process and cross validating it with physiological and biochemical markers… Time point analysis was performed because use of single time point can lead to static outcomes. This all can comprehensively help understand the acclimatization physiology at 4,111m.

## Research design and methods

### Human volunteers and study design

The population group under study consists of young male residents of Kyrgyzstan (≤800m) origin (n = 30) matched for age and BMI. Volunteers were fresh inductees to high altitude, did not show any kind of cardiovascular or neurological disease at basal as per their medical record data and were not into any kind of medication or supplementation. Volunteers were provided the same food/ration *ad libitum* with similar nutritional aspects during entire study duration. Protocol of the study was approved and carried out according to the guidelines by Ethics Committee of Defence Institute of Physiology and Allied Science (DIPAS), Delhi. After explaining the study protocol written informed consent was obtained from each individual. Basal studies started at Bishkek, Kyrgyzstan (800 m) and data was collected. Volunteers were inducted by road to ~3000m (for 4 days) for acclimatization as per prescribed protocol of the study. After that, volunteers were moved to 4,111m by road where they stayed for 21 days and throughout the altitude stay temporal analysis was performed at Day3 (HAD3), Day7 (HAD7), Day14 (HAD14) and Day21 (HAD21). The basic characteristics of the individuals selected for the study has been provided in the Table 1.

**Table 1:** Volunteers basic characteristics.

Characteristics	Kyrgyz (n = 30)
Age, year	
Median	20.5
Interquartile range	19–23
BMI, Kg/m^2^	
Median	23.5
Interquartile range	21.6–24.7
Barometric pressure (mmHg)	
Basal	692
High Altitude time points	477
Mode of induction to altitude	By road
Other information	
Previous exposure to high altitude	No
Smoker/ Drinker	No, except 2 individuals which were abstained to smoke/drink during study protocol
History of cardiovascular and neurological disease	No

### Lake Louise Acute Mountain Sickness score recording

A very straightforward and common grading system for diagnosis of acute mountain sickness is the Lake Louise self-assessment questionnaire [15] with a headache and a score of ≥3 (ranges 0 to 12) represent AMS. The standard Lake Louise symptom questionnaire includes assessments like headache, gastrointestinal symptoms, fatigue and dizziness to produce a total score between 0 and 12. Each symptom was scored on a scale between 0 and 3 by volunteers (0 = none; 1 = mild; 2 = moderate; 3 = severe). Before initial recording volunteers were introduced with the terminologies, meaning of each symptom and how to identify signs of AMS and symptoms of AMS appeared in volunteers after ascent to high altitude were recorded. If volunteers had question regarding the meaning of the symptoms, question was rephrased and provided with an example to make them understand. AMS recording was done on first 7 days at 4,111m altitude stay.

### Physiological measurements

#### Blood pressure, heart rate, arterial oxygen saturation, body weight and height

Systolic and diastolic blood pressure along with heart rate was measured using mercury free BP instrument (OMRON, USA) in the morning hours (0600–0700 hrs) in supine position when the participants were awake but still lying on bed. Saturation of peripheral oxygen was measured using finger pulse oximeter (Nonin Medical Inc, USA). It specifically measures percentage of oxygenated hemoglobin compared to total hemoglobin in blood giving an estimate of arterial oxygen saturation. Body mass was recorded after voiding between 0600–0800 hrs before breakfast using electronic platform balance (Deca 770, Seca incorporation, USA) and height was recorded using calibrated height rod (Seca Ltd, Medical Scale and measuring system, Brimingham, UK; Least Count:1 mm).

#### Pulmonary artery pressure by two-dimensional Doppler echocardiography

Two-dimensional and Doppler echocardiography were performed from the standard parasternal, apical, and subcostal views in the resting state, in the supine or left lateral position using ultrasound systems (Phillips SX50, with transducer S5-1, Bothell, USA). The standard M-mode measurements of aorta, left atrium, left and right ventricular wall thickness, and left ventricular end-diastolic and systolic dimensions and right ventricular dimension at end-diastole and end-systole were made from the parasternal long-axis view as recommended by the American Society of Echocardiography [16]. All reported values represent the mean of at least 3 measurements. Peak E and A diastolic inflow velocities from mitral and tricuspid valves and tissue Doppler imaging (TDI) derived velocities of lateral and septal mitral annulus and lateral tricuspid annulus were measured to characterize left and right ventricular diastolic functions. Systolic pulmonary artery pressure (SPAP) was estimated using maximal tricuspid regurgitation (TR) jet velocity (V), assessed by continuous Doppler and transformed by the modified Bernoulli equation (4* V^2^) to which right atrial pressure (RAP) was added. RAP was considered as equal to 5 mmHg, taking into account that all volunteers displayed IVC < 2 1 mm and > 50% collapsibility. Mean pulmonary artery pressure (mPAP) can be approximated from the SPAP, using the following formula = 0.6 * SPAP + 2mmHg [17]. These calculations were based on multiple TR velocity measurements in nearly all cases (median number of TR velocity measurements = 6).

### Blood collection; serum, plasma separation and storage

Blood samples were collected at basal, high altitude day 3, day 7, day 14 and day 21 between 0600–0800 hrs from anticubital vein after 12 hours fasting in ethelnediaminetetraacetic acid (EDTA) and gel tubes at basal (control) and at different altitude time points (test). The tubes were centrifuged; plasma and serum were separated by centrifugation at 3000 rpm for 20 min at 4°C and stored at -80°C until assayed. Venous blood samples from individuals at sea level and at different altitude time points was also collected in PAX gene Blood RNA Tubes (PAXgene, PreAnalytix, Hombrehtikon, Switzerland, distributed by Qiagen, catalogue no. 762165). PAX contains 6.5 ml of RNA stabilizing solution in which 2.5 ml of whole blood is added using a blood collection accessory at room temperature. After blood collection, PAX tube was inverted 8–10 times, were kept upright at room temperature (18–25°C) for 2 hours than transferred to freezer (-20°C) for 24 hours and finally stored at -80°C.

### RNA processing

#### RNA isolation, quantification and quality check

Isolation of total cellular RNA was done using PAX gene blood RNA Kit (PreAnalytix) as per manufacturer’s protocol. DNase digestion was carried out for the removal of DNA using RNase free DNase set (Qiagen) on the RNA spin column. Purified RNA was immediately chilled on ice. RNA concentration and quality was evaluated by measuring absorbance at 260 and 280 nm using a spectrophotometer (NanoDrop, USA) before being stored at -80°C. The RNA samples were quantified using Qubit HS RNA kit (which specifically quantitates RNA). The samples were checked for degradation using Agilent Bioanalyzer RNA 6000 nano kit for determination of RNA Integrity Number (RIN) with 1 being the most degraded and 10 being the least, calculated by computing the ratio of the areas under 18S and 28S rRNA peaks, the total area under the graph and by measuring the height of 28S peak. The RIN values more than 7 were considered for further analysis.

#### RNA sequencing and data availability

Two technical replicate samples for each control i.e. Basal and test i.e. high altitude D3, D7, D14 and D21 were transferred to Illumina HiseqX sequencing system and loaded sequencing by synthesis (SBS) reagents as per Illumina recommendation to perform 2x150 paired end sequencing. There were approximately 45 million (150-nt length) reads. The median quality score was >30 across all the samples. The sequence reads were mapped to the Human reference genome (HG19) with STAR aligner (V.2.0). The STAR mapped reads were processed to remove PCR and optical duplicates using Picard tools and raw expression count was generated using feature Counts. The RNA sequencing data is submitted to Gene Expression Omnibus (GEO): Accession Number: GSE133702.

#### RNA sequencing data normalization

The sequence data quality was checked using FastQC and MultiQC software. The data was checked for base call quality distribution, % bases above Q20, Q30, %GC, and sequencing adapter contamination. All the samples in technical replicates have passed QC threshold (Q30>80%). Raw sequence reads were processed to remove adapter sequences and low quality bases using Trimgalore. The QC passed reads were mapped onto indexed Human reference genome (HG19) using STAR v2 aligner. Overall ~94.56% of the reads aligned onto the reference genome. The PCR and optical duplicates were marked and removed using Picard tools. Gene level expression values were obtained as read counts using featureCounts software. Expression similarity between biological replicates was tested by spearman correlation. For differential expression analysis the biological replicates were grouped as test (the altitude time points group) and control (the basal group). Differential expression analysis was carried out using edgeR package. The read counts were normalized and 40509 features (70.06%) have been removed from the analysis because they did not have at least 1 counts-per-million in at least 2 samples.

### Differentially expressed gene analysis

#### Differentially expressed genes data representation

Gene expression pattern signal intensities of each gene probe were acquired by clustering according to the Euclidean distance. It reveals the correlation during temporal analysis in Kyrgyz for each gene probe to generate heat map in which matrix of values is mapped with matrix of colors. Heat map was constructed using web based matrix visualization and analysis platform Morpheus for group wise analysis [18] and CimMiner for sample wise analysis (http://discover.nci.nih.gov/cimminer). Venn diagram representation of differentially expressed genes in Kyrgyz was done using ‘InteractiVenn’ [19].

#### Gene enrichment analysis using DAVID, functional annotation clustering using Morpheus and Gene Ontology analysis using cytoscape BinGO plugin

The differentially expressed genes were studied for their overabundance in different GO terms as well as pathways. The overabundance of a particular term was decided based on the number of significant genes in the analysis. Functional annotation clustering was achieved using the database for annotation, visualization and integrated discovery (DAVID) (https://david.ncifcrf.gov/) can provide systematic and comprehensive biological function annotation information for high throughput gene expression. Therefore, we applied KEGG pathway analyses to the DEGs using DAVID online tool at the functional level. Differentially expressed genes which were segregated both numerically log2FC≥ ±0.58 and significantly pvalue <0.05 were assigned for official gene number and accession number. These genes were put into DAVID analysis based on official gene name and accession number to extract KEGG enrichment. Pathways obtained for the enriched genes were ranked based on the adjusted p value (Benjamini- Hochberg adjustments). The identified gene ontologies were screened using Morpheus for their expression at different altitude time points. The file was uploaded to the Morpheus heat map building tool available from the Broad Institute. The file was formatted to conform to the expected column headings as described in the input file documentation. Custom colors were selected to represent high expression (red), moderate expression (pink), and low expression (blue). 394 common genes during temporal analysis were matched for the similar gene transcripts obtained from functional annotation clustering (using Morpheus) and those common genes were analysed for gene ontology using BinGO. Overrepresentation and visualization of gene ontology (GO) terms and construction of enrichment GO network using Cytoscape 3.2.1 with Biological Networks Gene Ontology tool BinGO plug-in. Node size represented number of targets and color represents significance of the GO category.

#### cDNA conversion of RNA, quantitative and technical validation of genes using real time quantitative reverse transcription-polymerase chain reaction

Technical validation was performed using samples from basal and high altitude day3 time point group. Samples for the technical validation were kept same as taken for the RNA sequencing with RNA integrity number >7. 1μg of RNA was converted into double stranded cDNA by reverse transcription using cDNA Synthesis Kit (RT^2^ First strand kit, Qiagen, USA) according to the manufacturers protocol with an oligo (dT) primer containing a T7 RNA polymerase site added 3’ of the poly (T). The selection of Gene of interest (GOI) was to validate the RNA sequencing data. The genes selected were considered because they were significantly differentially expressed and are involved in pathways obtained from DAVID analysis. The GOI’s are: TBXA2R, GP9, ITGB2, HBA1, VEGFB and TNF. The primers used for technical validation of GOI’s are shown in Table 2. Two steps qRT-PCR (Applied Biosystems, Thermo Fisher Scientific Corporation, USA) was performed to confirm the differential expression results obtained from the RNA sequencing experiments. Primers were designed for 7 genes and polymerase chain reaction conditions were optimized individually using SYBR green (Qiagen, Hilden, Germany). The quantitative RT-PCR was run with SYBR green mix in a final volume of 25μl. The PCR thermo-cycler program was set at on Step one Plus software for 95°C for 10 minutes followed by 40 cycles of 95°C for 15sec, 55°C for 60 seconds. Melt curve analysis followed the PCR step. Each RTPCR experiment was replicated before final data analysis for final interpretation. 2^-ΔΔCT formula was used for determination of the fold change in each GOI. Endogenous control used was 18s for the normalization of gene of interest expression values.

**Table 2:** Primers designed used for technical validation of gene expression by real time PCR.

GENE SYMBOL	FORWARD PRIMER (5’-3’)	REVERSE PRIMER (5’-3’)
TBXA2R	TGTCCTTCCTGCTGAACACG	ACTGTCTGGGCGATGAAGAC
GP9	TGTATCCCATAGAGTTGCCACC	TCTCTGGACACCGTAGAAGG
ITGA2B	ACGCATGGTTCAACGTGTC	ATTGGAATCGCCCTCTCCTC
VEGFB	GCTGACATCACCCATCCCAC	AGTCACCCTGCTGAGTCTGA
HBA1	AACTTCAAGCTCCTAAGCCA	CAGGAACTTGTCCAGGGAG
TNF	AAGCCTGTAGCCCATGTTGT	TATCTCTCAGCTCCACGCCA
18s	GGACATCTAAGGGCATCACAG	TCAAGAACGAAAGTCGGAGGTT

#### Network analysis of the Gene coexpression using cytoscape GeneMANIA plugin and identification of significant hub genes

GeneMANIA cytoscape plugin, which is an open resource platform for visualizing complex networks, was used to establish gene interaction relationship network, gene coexpression and hub genes were identified. Time point day 3 differentially expressed genes in comparison to basal were taken for analysis. Gene showing coexpression network analysis from cytoscape was done using five calculation methods: Average shortest path length, degree, clustering coefficient, closeness centrality and betweenness centrality. The intersecting genes derived using these five algorithms encodes core proteins and may represent key candidate genes with important biological regulatory function.

### Biochemical analysis for biological validation

In accordance with the physiological, DAVID and network analysis expression changes we determined quantitatively proteins showing involvement in significantly perturbed biological pathways involved at high altitude. Angiotensin Converting enzyme (ACE, R&D systems, USA), Cortisol (DBC, Canada), Coagulation Factor VIII (F8, eLabscience, USA), Vascular endothelial growth factor (VEGF, R&D systems, USA) and Vimentin (VIM, eLabscience, USA) were determined at basal, high altitude D3, D7, D14 and D21 according to manufacturer’s protocol.

### Statistical analysis

Physiological measurements were represented as mean±SEM in graph. For RNA sequencing analysis, the final set of differentially expressed genes shown in S1 File was filtered based on statistical significance of adjusted p value of <0.05 and log2 fold change ≥±0.58 in comparison to basal for each time point. For the functional enrichment analysis the genes with considerably enriched GO terms in DEG’s were selected statistically by Bonferroni/ Benjamini-hochberg FDR correction. Biochemical analysis for biological validation of gene expression was represented as mean±SEM and statistically analyzed using student t-test. A p value of ≤0.05 was applied as the cut off value for statistical significance at all time.

## Results

**Fig 1:**
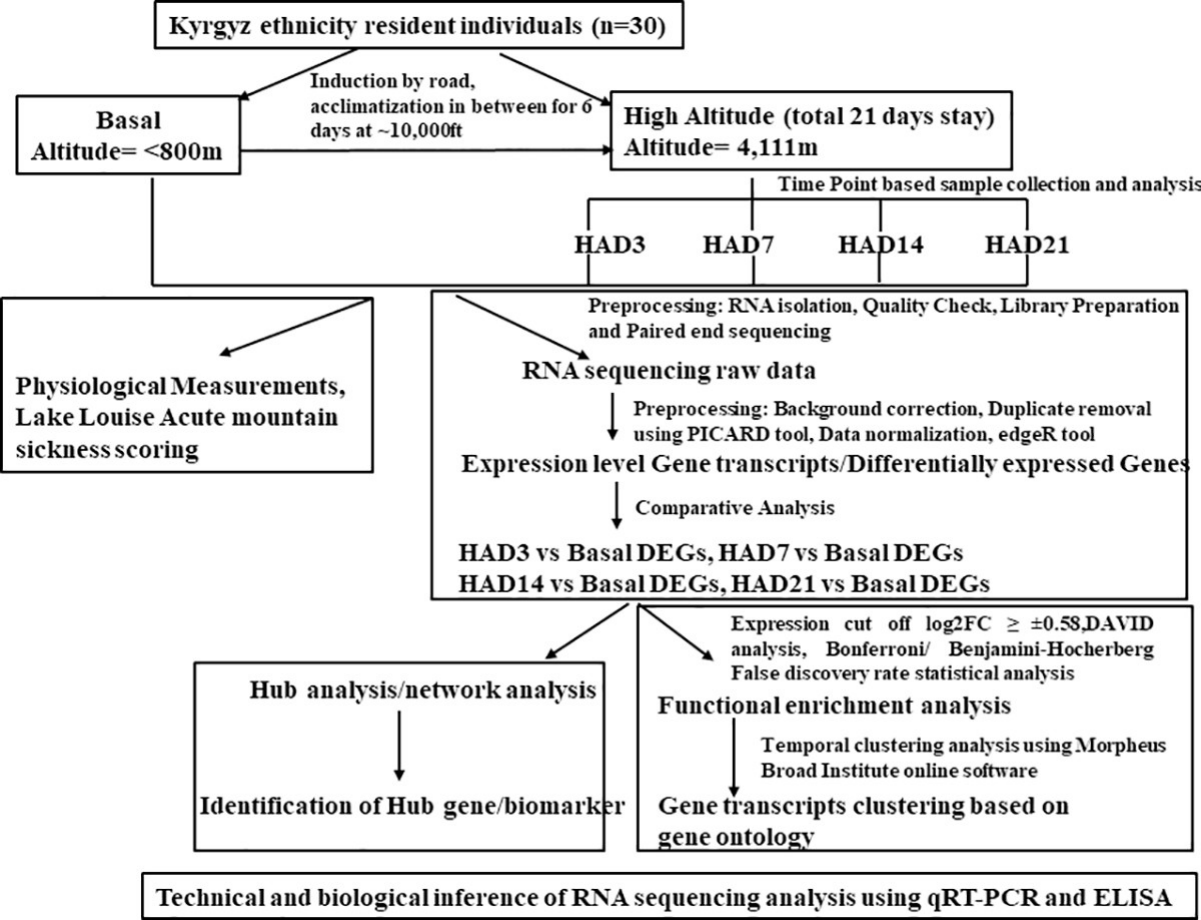
Schematic representation of the work flow approach undertaken for the high altitude temporal analysis. It includes: Sample collection, data collection, pre-processing, analysis and validation of the data.

To comprehend the complex response towards high altitude hypoxia we studied physiological perturbation in Kyrgyz population along with whole genomic changes in temporal manner. We designed experiment strategies adopted for the current study as schematically summarized and represented in Fig 1.

### Time point study at high altitude present considerable effect on physiological parameters

**Fig 2:**
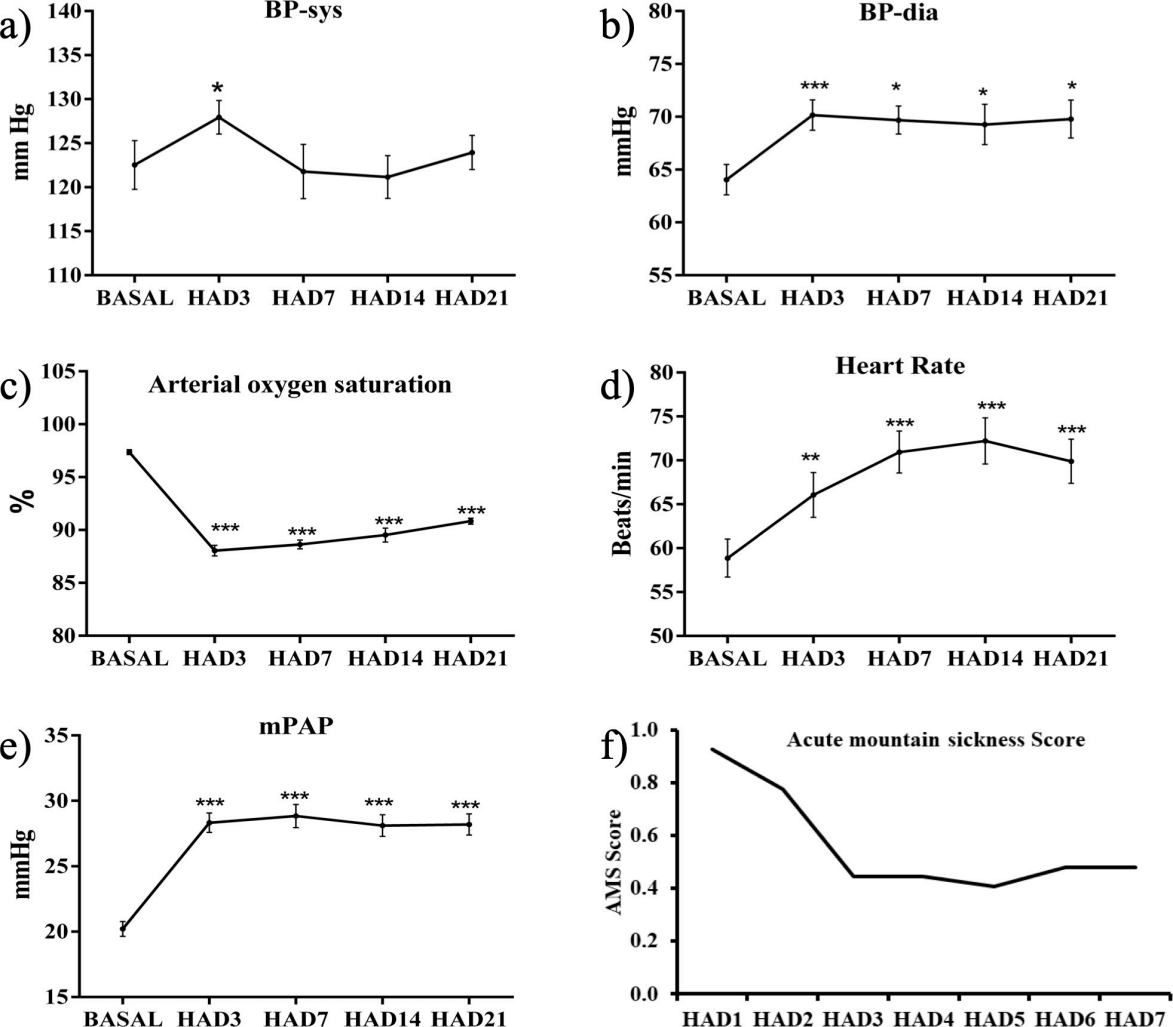
Line graph showing changes mean±SEM of 30 individuals at basal and different high altitude time points HAD3, HAD7, HAD14, and HAD21 for (**a-e**) BP-sys, BP-dia, Heart Rate, Arterial oxygen saturation, and mPAP. Statistical significance is represented as *p<0.05, **p<0.01, and ***p<0.001 at different high altitude time points in comparison to basal. (**f**) Represents the AMS scoring obtained at first 7 high altitude days of 30 individuals. Abbreviations: HAD3, high altitude day 3; HAD7, high altitude day 7; HAD14, high altitude day 14; HA21, high altitude day 21; BP-sys, systolic blood pressure; BP-dia, diastolic blood pressure; mPAP, mean pulmonary artery pressure; AMS, Acute Mountain Sickness.

To determine the severity of high altitude hypoxic stress during different time points investigations from our sample size of n = 30 were done. Physiological parameters that are directly related to high altitude condition like blood pressure, heart rate, arterial oxygen saturation, mean pulmonary artery pressure and acute mountain sickness score were investigated. Systolic blood pressure increased significantly on D3 (p<0.05) of HA as compared to basal. Thereafter, it reduced to almost basal level on D14 and D21 of high altitude (Fig 2A). Diastolic blood pressure increased upon ascent to high altitude on day3 (p<0.001) and remained almost similar on rest of the days at altitude (Fig 2B). Resting heart rate increased at HA D3, D7, and D14. At D3 increase was statistically significant as compared to basal (p<0.01) and heart rate increased at later time points significantly D7 & 14 (p<0.001). On D21 there was slight drop in heart rate but as compared to basal the increase was significant (p<0.01) (Fig 2C). Oxygen saturation in blood decreased to a great extent on HAD3 as compared to basal, thereafter it regained but remained significantly less than basal at each time point (p<0.001) (Fig 2D). Mean pulmonary artery pressure increased with altitude exposure. The significant up regulation (p<0.001) is observed at all-time points (Fig 2E). We investigated the prominence of responses individual recognized on reaching to high altitude as compared to basal. Acute mountain sickness prevalence was recorded at initial 7 days of altitude. To determine the severity of stress, investigations from our sample size of individuals was done, the individual and mean score remained less than 3, indicating no prevalence of AMS (Fig 2F). The sequential changes in physiology i.e. BP (systolic and diastolic), HR, SpO_2_, pulmonary artery pressure & AMS scoring allowed us to capture the transition between early high altitude exposed physiological changes and later stable response.

### Data acquiring and analysis of differentially expressed genes at different high altitude time point in comparison to basal

**Fig 3:**
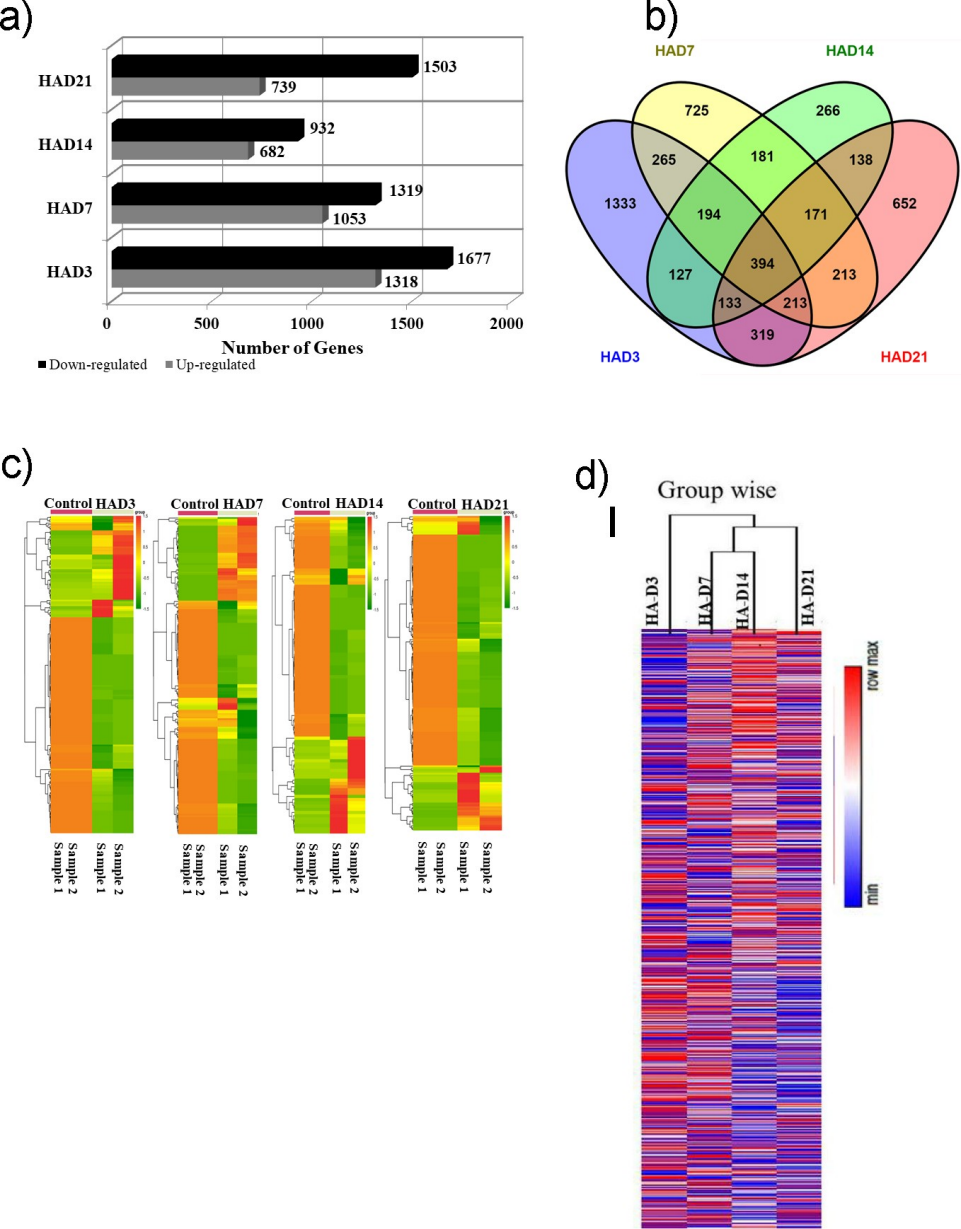
(**a**) Differentially expressed genes represented as bar graph–comparative up regulated and down regulated bars of all high altitude time points in comparison to basal (**b**) Venn diagram show the overlap of high altitude time series DEGs in comparison to basal. Overlap is shown among and between all high altitude time points. (**c**) Hierarchical clustering of the differentially expressed genes depicting 4 high altitude time points as compared with control. Expression values of specific genes (in technical replicates) are represented by colour intensities as shown in the reference colour key. Euclidean distance measure hierarchical clustering algorithm was used. Heat map was constructed online using CIMiner tool. (**d**) Group wise hierarchical clustering of the differentially expressed genes depicting 4 high altitude time points. Expression values of specific genes are represented by colour intensities as shown in the reference colour key. Abbreviations: HAD3, high altitude day 3; HAD7, high altitude day 7; HAD14, high altitude day 14; HAD21, high altitude day 21.

The changes in physiological markers at early time point directed our attention in deciphering the underlying molecular basis of systemic changes involved during different time point in Kyrgyz. We further generated and investigated the changes utilizing RNA sequencing from whole blood RNA post high altitude exposure during each time point. Transcriptome data sets of different time points were assessed after applying cut off of Log2FC≥±0.58. During different time points of high altitude hypoxia exposure the number of genes upregulated and downregulated was represented as bar graph (Fig 3A). At HAD3 up gene count: 1318 and down: 1677 which is maximum number of perturbed genes among all time points. Later, at HAD7 the gene count for up: 1053 and down: 1319; HAD14 up: 682 and down: 932 and at HAD21 up: 739, down: 1503. To understand the temporal pattern of mRNA, we investigated overlapping gene by Venn diagram (Fig 3B). It showed all possible logical relations between finite significant time-point gene sets that showed 394 genes as common to all 4 sets of post hypoxic exposure when compared with basal. At individual time point maximum genes perturbed was observed at HAD3 i.e. 1333 genes. Further, in rest time points, HAD7: 725 genes; HAD14: 266 genes; HAD21: 652 genes were found to be perturbed uniquely. These genes were differentially expressed in comparison to basal at each time point. We also performed the unsupervised learning by applying hierarchical clustering sample wise for two technical replicate (Fig 3C) and group wise (Fig 3D) to infer the data of the expression profiles for genes across the temporal analysis in Kyrgyz. The hierarchical clustering of differentially expressed genes data set in samples of different time point in Kyrgyz as compared to basal noticeably separated HAD3 time point from rest of the time point analysis. We infer that this enriched gene clustering of samples is indicative of unique and maximum gene expression in Kyrgyz at HAD3. This investigation also goes in line with unsupervised hierarchical clustering; indicating clearly the unique nature of D3 exposed group Kyrgyz individuals when compared to basal. Taken together, this experiment indicates unique underlying biological phenomenon of early time point HAD3.

### DAVID Bioinformatics of differentially regulated genes in Kyrgyz at different time points of high altitude exposure

To integrate the complex physiological analysis and transcriptomic data clustering of differentially expressed genes, DAVID Bioinformatics was performed. Transcriptomic data sets of different time points were assessed after applying cut off of Log2FC≥0.58. Up regulated genes and down regulated genes were considered for finding out the considerably enriched KEGG pathways (Table 3). Maximum pathways appeared from DAVID analysis was at HAD3 time point. Also, it was clear from DEG data that maximum number of genes been perturbed at HAD3 when compared to basal. Total (upregulated and downregulated) pathways obtained were, at HAD3: 31; HAD7: 15; HAD14: 12; HAD21: 19. It is clear that maximum genes were involved at HAD3 acclimatization response in Kyrgyz individuals which are actually demonstrating the initial appearance of physiological changes as seen in HR, BP, SpO_2_, and mPAP. Some of the critical pathways with highest genes involved are: HAD3: Cell adhesion molecules (gene count: 31), Axon guidance (Gene count: 25); MAPK signalling (Gene count: 22), Rap1 signalling (Gene count: 21), Endocytosis (Gene Count: 21), Purine metabolism (Gene count: 15). HAD7: Cell adhesion molecules (Gene Count: 21); Ribosome (Gene count: 14), cytokine-cytokine receptor interaction (Gene count: 14), Axon guidance (Gene count: 11), Hematopoietic cell lineage (gene count: 7). HAD14: Cell adhesion molecules (Gene count: 9); Hematopoietic cell lineage (gene count: 9), Gap junction (gene count: 8), Platelet activation (Gene count:7). HAD21: Ribosome (Gene count: 28); Rap1 signalling (Gene count: 14); oxidative phosphorylation (Gene count: 11); upregulated pathways: cytokine-cytokine receptor interaction (gene count: 13), PI3K-Akt signalling (Gene count: 12), chemokine signalling (Gene count: 11). Involvement of maximum genes and correspondingly maximum pathways at HAD3 time point leads the way that initial time point is very critical for understanding the pattern of acclimatization in high altitude induced hypoxic environment. Initial physiological changes like reduced SpO_2_, increased BP, HR and mPAP response could directly relate the perturbation of whole genome for acclimatization at HAD3. The response majorly covers the fact that there is a clear transition state in human body when reached to high altitude from basal and at later stage the response get stabilized.

**Table 3:** Listing downregulated and upregulated pathways, gene count, p value and gene symbol obtained from DAVID analysis of differentially expressed genes (logFC≥±0.58) at different high altitude time point’s D3, D7, D14 and D21 in comparison to basal.

S.NO	PATHWAY NAME (HAD3 *vs* BASAL)	COUNT	P VALUE	NO. OF GENES UP AND DOWN (GENE SYMBOL)
1	hsa04612:Antigen processing and presentation	15	7.40E-06 (up)	Up: 15/15 (TNF, CD8A, HLA-A, HSPA1A, KIR2DS4, HLA-C, HSPA1B, HLA-B, HLA-G, HSPA1L, KIR2DL1, KIR2DL3, KIR3DL1, KIR2DL4, KIR3DL2)
2	hsa04640:Hematopoietic cell lineage	13	4.67E-04 (up)	Up: 13/13 (TNF, CD8A, CD3E, CSF1, FCER2, GP9, CD55, CD19, GP1BB, CD22, CD14, CD7, ITGA2B)
3	hsa05412:Arrhythmogenic right ventricular cardiomyopathy	22	3.55E-04(down), 0.0049(up)	Down: 12/22(CACNA2D1, PKP2, ITGAV, DMD, CACNB2, CACNB4, ITGA4, CDH2, CACNA1D, TCF7L2, CTNNA3, CTNNA2) Up: 10/22 (ACTB, ACTG1, CACNG8, ITGB7, CACNG6, LMNA, DSP, CACNB3, CACNA2D2, ITGA2B)
4	hsa05416:Viral myocarditis	10	0.001 (up)	Up: 10/10 (ACTB, ACTG1, PRF1, CD55, RAC2, HLA-A, HLA-C, ITGB2, HLA-B, HLA-G)
5	hsa04015:Rap1 signalling pathway	21	0.0015 (up)	Up: 21/21 (ACTB, FGFR1, TLN1, FLT4, MAP2K3, CSF1, SIPA1, EFNA3, FPR1, ITGB2, LPAR2, RALGDS, ACTG1, VEGFB, LAT, PFN1, RAC2, LPAR5, RRAS, EFNA4, ITGA2B)
6	hsa04145:Phagosome	17	0.0017 (up)	Up: 17/17 (ACTB, TUBB2B, TUBB2A, HLA-A, HLA-C, ITGB2, HLA-B, HLA-G, ACTG1, TUBA8, CORO1A, TUBA4A, ATP6V0D1, TUBA1A, CD14, TUBB4A, TUBB4B)
7	hsa04514:Cell adhesion molecules	31	0.002 (down), 0.055 (up)	Down: 16/31 (NRCAM, NCAM1, NCAM2, NRXN3, ITGAV, CD274, NLGN1, CNTNAP2, CDH2, ITGA4, NRXN1, CLDN20, LRRC4C, HLA-DQA2, CDH4, NEGR1) Up: 15/31 (CD8A, CLDN5, ICAM3, HLA-A, CD99, HLA-C, ITGB2, HLA-B, HLA-G, PDCD1, ITGB7, CD22, ESAM, SELPLG, ICOSLG)
8	hsa04360:Axon guidance	25	0.005 (down), 0.067 (up)	Down: 14/25 (DCC, NRP1, LRRC4C, NTN1, SLIT3, EPHA5, SEMA6A, EPHA6, PAK3, ROBO1, SRGAP3, SEMA3C, ROBO2, SRGAP1) Up: 11/25 (EPHB6, RAC2, EFNB1, ABLIM3, EFNA3, CFL1, EFNA4, ROBO3, EPHA1, EPHB4, SLIT1)
9	hsa04010:MAPK signalling pathway	22	0.0067 (up)	Up: 22/22 (PTPN7, FGFR1, TNF, CACNG8, MAP2K3, CACNG6, MKNK2, MAP4K1, CACNB3, HSPA1A, HSPA1B, CACNA2D2, FLNA, HSPA1L, FOS, RAC2, JUN, NTRK1, RRAS, MAPK7, DUSP8, CD14)
10	hsa05410:Hypertrophic cardiomyopathy (HCM)	10	0.009 (up)	Up: 10/10 (ACTB, ACTG1, TNF, CACNG8, ITGB7, CACNG6, LMNA, CACNB3, CACNA2D2, ITGA2B)
11	hsa00512:Mucin type O-Glycan biosynthesis	6	0.01 (down)	Down: 6/6 (GALNT3, WBSCR17, GALNTL6, GALNT18, GALNT13, C1GALT1)
12	hsa04940:Type I diabetes mellitus	7	0.012 (up)	Up: 7/7 (PRF1, TNF, HLA-A, HLA-C, HLA-B, HLA-G, LTA)
13	hsa05414:Dilated cardiomyopathy	10	0.015 (up)	Up: 10/10 (ACTB, ACTG1, TNF, CACNG8, ITGB7, CACNG6, LMNA, CACNB3, CACNA2D2, ITGA2B)
14	hsa04144:Endocytosis	21	0.015 (up)	Up: 21/21 (DNM3, HLA-A, CXCR1, HLA-C, HSPA1A, HSPA1B, HLA-B, HLA-G, HSPA1L, RAB11FIP5, ARPC1B, ADRB2, RAB11FIP3, FOLR3, FOLR2, NTRK1, SNX32, BIN1, EHD1, AGAP2, EHD3)
15	hsa05332:Graft-versus-host disease	6	0.016 (up)	Up: 6/6 (PRF1, TNF, HLA-A, HLA-C, HLA-B, HLA-G)
16	hsa05143:African trypanosomiasis	6	0.016 (up)	Up: 6/6 (TNF, HBA2, HBA1, HBB, HPR, TLR9)
17	hsa04380:Osteoclast differentiation	13	0.017 (up)	Up: 13/13 (FOS, TNF, LILRA2, LILRA3, SOCS3, JUN, LILRA4, CSF1, LILRA5, OSCAR, LILRA6, FOSB, SIRPA)
18	hsa05340:Primary immunodeficiency	6	0.019 9 (up)	Up: 6/6 (ORAI1, CD19, CD8A, CD3E, CD79A, ADA)
19	hsa05323:Rheumatoid arthritis	10	0.019 (up)	Up: 10/10 (FOS, CCL3, TNF, CXCL5, CCL3L1, CSF1, JUN, ITGB2, CCL5, ATP6V0D1)
20	hsa04724:Glutamatergic synapse	12	0.02 (down)	Down: 12/12 (GRM5, TRPC1, GRIN2B, GRIK1, GRM8, GRIK2, GRM7, GRIK4, GNB4, PLA2G4C, CACNA1D, SHANK2)
21	hsa04512:ECM-receptor interaction	20	0.02(down); 0.046 (up)	Down: 10/20: (LAMA2, COL4A3, CD36, ITGAV, RELN, ITGA4, COL5A3, COL24A1, SPP1, THBS4) Up: 9/20 (LAMA5, GP1BB, ITGB7, COL6A3, COL6A2, SV2A, LAMC1, GP9, ITGA2B)
22	hsa05330:Allograft rejection	6	0.026 (up)	Up: 6/6 (PRF1, TNF, HLA-A, HLA-C, HLA-B, HLA-G)
23	hsa04510:Focal adhesion	17	0.027 (up)	Up: 17/17 (ACTB, TLN1, FLT4, FLNA, ACTG1, VEGFB, RAC2, LAMA5, ITGB7, JUN, COL6A3, COL6A2, LAMC1, ZYX, PARVB, MYLK, ITGA2B)
24	hsa00230:Purine metabolism	15	0.03 (down)	Down: 15/15 (ADPRM, POLR2K, PNPT1, PDE11A, PDE10A, PDE4D, AK5, PPAT, PDE7B, PDE1C, ADK, RRM2, GUCY1A2, PDE9A, ADCY10)
25	hsa04662:B cell receptor signalling pathway	8	0.038 (up)	Up: 8/8 (FOS, CD19, RAC2, JUN, CD81, CD22, CD79B, CD79A)
26	hsa03010:Ribosome	12	0.048 (down)	Down: 12/12 (RPL17, RPS26, MRPL22, MRPL13, MRPS18C, MRPL15, RPS3A, RPL9, RPL34, RPS15A, RSL24D1, RPS7)
27	hsa04650:Natural killer cell mediated cytotoxicity	11	0.054 (up)	Up: 11/11 (PRF1, LAT, TNF, RAC2, ARAF, KIR2DL1, ITGB2, KIR2DS4, KIR2DL3, KIR2DL4, HCST)
28	hsa04620:Toll-like receptor signalling pathway	10	0.054 (up)	Up: 10/10 (FOS, CCL3, TNF, CCL3L1, JUN, MAP2K3, TLR3, CCL5, CD14, TLR9)
29	hsa04122:Sulfur relay system	3	0.074 (up)	Up: 3/3 (TST, CTU2, MOCS1)
30	hsa00480:Glutathione metabolism	6	0.08 (down)	Down: 6/6 (GSTM1, LAP3, GSTM2, GSTM3, RRM2, GSTM5)
31	hsa04390:Hippo signaling pathway	12	0.088 (up)	Up: 12/12 (ACTB, DVL2, ACTG1, PPP2R1A, WNT10B, YWHAH, CRB2, RASSF1, TEAD2, ITGB2, LLGL2, AXIN1)
**S.NO**	**PATHWAY NAME (HAD7 *vs* BASAL)**	**COUNT**	**P VALUE**	**NO. OF GENES UP AND DOWN (GENE SYMBOL)**
1	hsa03010:Ribosome	14	5.17E-04 (down)	Down: 14/14 (RPS26, RPS18, RPS27, RPL23, RPS17, RPL35, RPL27, RPS15A, RPL36, RPL5, RPL38, RPL39, RPS8, RPL29)
2	hsa04610:Complement and coagulation cascades	8	0.0073 (down)	Down: 8/8 (C1QB, CR1, F5, C3, CFB, SERPING1, C4BPA, C2)
3	hsa04360:Axon guidance	11	0.014 (up)	Up: 11/11 (SEMA5A, PAK6, DCC, SEMA6D, ABLIM3, SEMA7A, EFNA5, LRRC4C, EPHB4, SLIT1, SLIT3)
4	hsa04514:Cell adhesion molecules (CAMs	21	0.019 (down); 0.062 (up)	Down: 11/21 (NCAM1, LRRC4, SIGLEC1, ICOS, CD58, CD274, VCAN, NLGN3, CDH2, HLA-DQA2, SDC3) Up: 10/21 (NCAM2, NRXN3, CLDN5, NLGN1, CD99, ESAM, HLA-C, NEO1, LRRC4C, JAM3)
5	hsa00480:Glutathione metabolism	6	0.026 (down)	Down: 6/6 (GSTM1, LAP3, GSTM2, GSTM4, RRM2, ANPEP)
6	hsa00532:Glycosaminoglycan biosynthesis—chondroitin sulfate / dermatan sulfate	4	0.031 (up)	Up: 4/4 (CSGALNACT1, CHST7, B3GALT6, CHST14)
7	hsa04060:Cytokine-cytokine receptor interaction	14	0.041 (down)	Down:14/14 (CCR9, IFNAR2, TNFSF10, IL2RA, FLT3, CCR1, TNFRSF13B, CCL4L1, TNFRSF17, TNFSF14, CCL4L2, EDAR, PF4V1, TNFSF8)
8	hsa04122:Sulfur relay system	3	0.046 (up)	Up: 3/3 (TST, CTU1, MOCS1)
9	hsa00240:Pyrimidine metabolism	8	0.056 (down)	Down: 8/8 (TYMS, UPB1, POLR2J, RRM2, ENTPD1, POLR2J3, POLR2A, CMPK2)
10	hsa04672:Intestinal immune network for IgA production	5	0.068 (down)	Down: 5/5 (CCR9, ICOS, TNFRSF13B, TNFRSF17, HLA-DQA2)
11	hsa04924:Renin secretion	6	0.074 (up)	Up: 6/6 (KCNMA1, ACE, ADRB2, PDE1C, GUCY1A2, GUCY1B3)
12	hsa04640:Hematopoietic cell lineage	7	0.078 (up)	Up: 7/7 (CD19, IL9R, GP1BB, CD1C, CD14, GP9, ITGA2B)
13	hsa00500:Starch and sucrose metabolism	4	0.094 (down)	Down: 4/4 HKDC1, AMY1C, AMY2A, AMY1A
14	hsa00512:Mucin type O-Glycan biosynthesis	4	0.094 (up)	Up: 4/4 (GCNT3, WBSCR17, GALNTL6, GALNT13)
15	hsa00330:Arginine and Proline metabolism	5	0.098 (up)	Up:5/5 (PYCRL, SMOX, GAMT, AGMAT, CKB)
**S.NO**	**PATHWAY NAME (HAD14 *vs* BASAL)**	**COUNT**	**P VALUE**	**NO. OF GENES UP AND DOWN (GENE SYMBOL)**
1	hsa04640:Hematopoietic cell lineage	9	1.62E-04 (up)	Up: 9/9 (CD19, CR2, GP1BB, MS4A1, CD1C, CD22, CD14, GP9, ITGA2B)
2	hsa04540:Gap junction	8	0.0011 (up)	Up: 8/8 (TUBA8, TUBB2B, TUBB2A, GUCY1A2, TUBB6, GUCY1B3, PDGFC, TUBB4A)
3	hsa05202:Transcriptional misregulation in cancer	10	0.0039 (up)	Up: 10/10 (CEBPE, LYL1, MMP9, ELANE, PAX5, MPO, MEIS1, PTCRA, CD14, HIST1H3H)
4	hsa03010:Ribosome	9	0.005 (down)	Down: 9/9 (RPS26, RPS27, RPS29, RPS17, RPL36, RPL27, RPS15A, RPL39, RPS21)
5	hsa04514:Cell adhesion molecules (CAMs)	9	0.0067 (down)	Down: 9/9 (NCAM1, CADM1, CD274, NLGN2, CDH2, CLDN20, HLA-DQA2, CDH4, SDC3)
6	hsa04145:Phagosome	8	0.023 (up)	Up: 8/8 (TUBA8, TUBB2B, TUBB2A, MRC2, MPO, TUBB6, CD14, TUBB4A)
7	hsa04611:Platelet activation	7	0.033 (up)	Up: 7/7 (P2RY12, GP1BB, GUCY1A2, TBXA2R, GUCY1B3, GP9, ITGA2B)
8	hsa04662:B cell receptor signalling pathway	5	0.039 (up)	Up: 5/5 (CD19, CR2, CD22, CD79B, CD79A)
9	hsa02010:ABC transporters	4	0.055 (down)	Down: 4/4 (ABCA1, ABCB6, ABCA5, ABCG2)
10	hsa00520:Amino sugar and nucleotide sugar metabolism	4	0.068 (down)	Down: 4/4 HKDC1, GALE, CYB5RL, CHIT1
11	hsa04512:ECM-receptor interaction	5	0.077 (up)	Up: 5/5 (GP1BB, LAMC1, FN1, GP9, ITGA2B)
12	hsa04730:Long-term depression	4	0.099 (up)	Up: 4/4 (GNAZ, GUCY1A2, RYR1, GUCY1B3)
**S.NO**	**PATHWAY NAME (HAD21 *vs* BASAL)**	**COUNT**	**P VALUE**	**NO. OF GENES UP AND DOWN (GENE SYMBOL)**
1	hsa03010:Ribosome	28	5.02E-12(down)	Down: 28/28 (RPL35, RPL36, RPS15A, RPL37, RPL38, RPL39, RPS25, RPS26, RPS27, RPS28, RPS29, RPL34, FAU, RPS21, RPL36AL, RPL35A, RPL27, RPL24, RPL28, RPS18, RPS16, RPL23, RPL41, RPS17, RPS14, RPS13, RPL37A, RPS11)
2	hsa04612:Antigen processing and presentation	8	5.04E-04 (up)	Up: 8/8 (KLRC4, KLRC3, KIR2DL1, KIR2DL3, KLRD1, KIR3DL1, KIR2DL4, KIR3DL2)
3	hsa04060:Cytokine-cytokine receptor interaction	13	0.0012 (up)	Up: 13/13 (TNFRSF21, CCL3, IL2RB, TNFSF4, CXCR5, CCL3L1, FLT4, CCL3L3, CXCR6, CX3CR1, PDGFRB, CCL5, CCL28)
4	hsa05412:Arrhythmogenic right ventricular cardiomyopathy (ARVC)	10	0.0024 (down)	Down: 10/10 (CACNA2D1, PKP2, DMD, SGCD, CACNB4, CDH2, TCF7L2, TCF7L1, CTNNA3, CTNNA2)
5	hsa04062:Chemokine signalling pathway	11	0.0024 (up)	Up: 11/11 (GNGT2, CCL3, CXCR5, CXCL5, CCL3L1, CXCR6, CCL3L3, CX3CR1, CCL5, PIK3R3, CCL28)
6	hsa04360:Axon guidance	13	0.0059 (down)	Down: 13/13 (DCC, SEMA6C, EPHA6, SEMA6D, PAK3, ROBO1, SEMA3C, ROBO2, SEMA3A, LRRC4C, NTN1, SRGAP1, SLIT3)
7	hsa00564:Glycerophospholipid metabolism	6	0.014 (up)	Up: 6/6 (PNPLA7, PLA2G16, PLD4, DGKK, PCYT1B, GPAT2)
8	hsa00230:Purine metabolism	14	0.029 (down)	Down: 14/14 (POLR2F, POLR2L, POLR2J, PDE11A, PDE3A, PDE10A, POLR2J2, PDE6G, POLR2J3, POLE4, PDE1B, PDE1C, PDE9A, ADCY10)
9	hsa03020:RNA polymerase	5	0.041 (down)	Down: 5/5 (POLR2F, POLR2L, POLR2J, POLR2J2, POLR2J3)
10	hsa04932:Non-alcoholic fatty liver disease (NAFLD)	12	0.047 (down)	Down: 12/12 (NDUFB3, NDUFB4, NDUFS5, NDUFA3, UQCR11, CASP7, COX8A, NDUFA13, FAS, COX5B, NDUFA1, DDIT3)
11	hsa00190:Oxidative phosphorylation	11	0.047 (down)	Down: 11/11 (NDUFB3, NDUFB4, NDUFS5, NDUFA3, UQCR11, COX8A, NDUFA13, ATP5L, ATP5I, COX5B, NDUFA1)
12	hsa04620:Toll-like receptor signalling pathway	6	0.048 (up)	Up: 6/6 (CCL3, CCL3L1, CCL3L3, TLR3, CCL5, PIK3R3)
13	hsa04530:Tight junction	11	0.056 (down)	Down: 11/11 (SHROOM1, TJP1, OCLN, MYL5, MAGI2, MAGI1, RAB13, CLDN20, CTNNA3, CTNNA2, MYH10)
14	hsa04151:PI3K-Akt signalling pathway	12	0.057 (up)	Up: 12/12 (IL2RB, CDKN1A, GNGT2, CD19, LPAR5, FLT4, ITGB4, PDGFRB, TCL1A, LAMC1, PIK3R3, F2R)
15	hsa04610:Complement and coagulation cascades	7	0.064 (down)	Down: 7/7 (SERPINF2, CFB, SERPING1, C4BPA, C2, CFD, PROS1)
16	hsa04514:Cell adhesion molecules (CAMs)	11	0.068 (down)	Down: 11/11 (OCLN, NRXN3, CD274, NLGN1, HLA-DRB5, CDH2, CLDN20, LRRC4C, NEGR1, HLA-G, SDC3)
17	hsa04650:Natural killer cell mediated cytotoxicity	6	0.078 (up)	Up: 6/6 (PRF1, KIR2DL1, KIR2DL3, PIK3R3, KLRD1, KIR2DL4)
18	hsa04015:Rap1 signaling pathway	14	0.093 (down)	Down: 14/14 (EGFR, MAGI2, RAP1GAP, MAGI1, FGF14, FGF13, HGF, FGF12, DOCK4, PRKD1, GRIN2B, CNR1, RAPGEF5, ANGPT4)
19	hsa05202:Transcriptional misregulation in cancer	7	0.094 (up)	Up: 7/7 (PROM1, SLC45A3, IL2RB, CDKN1A, NTRK1, IGFBP3, HIST1H3H)

### Clustering the temporal expression of genes, biological pathways using Morpheus and BinGo analysis

**Fig 4:**
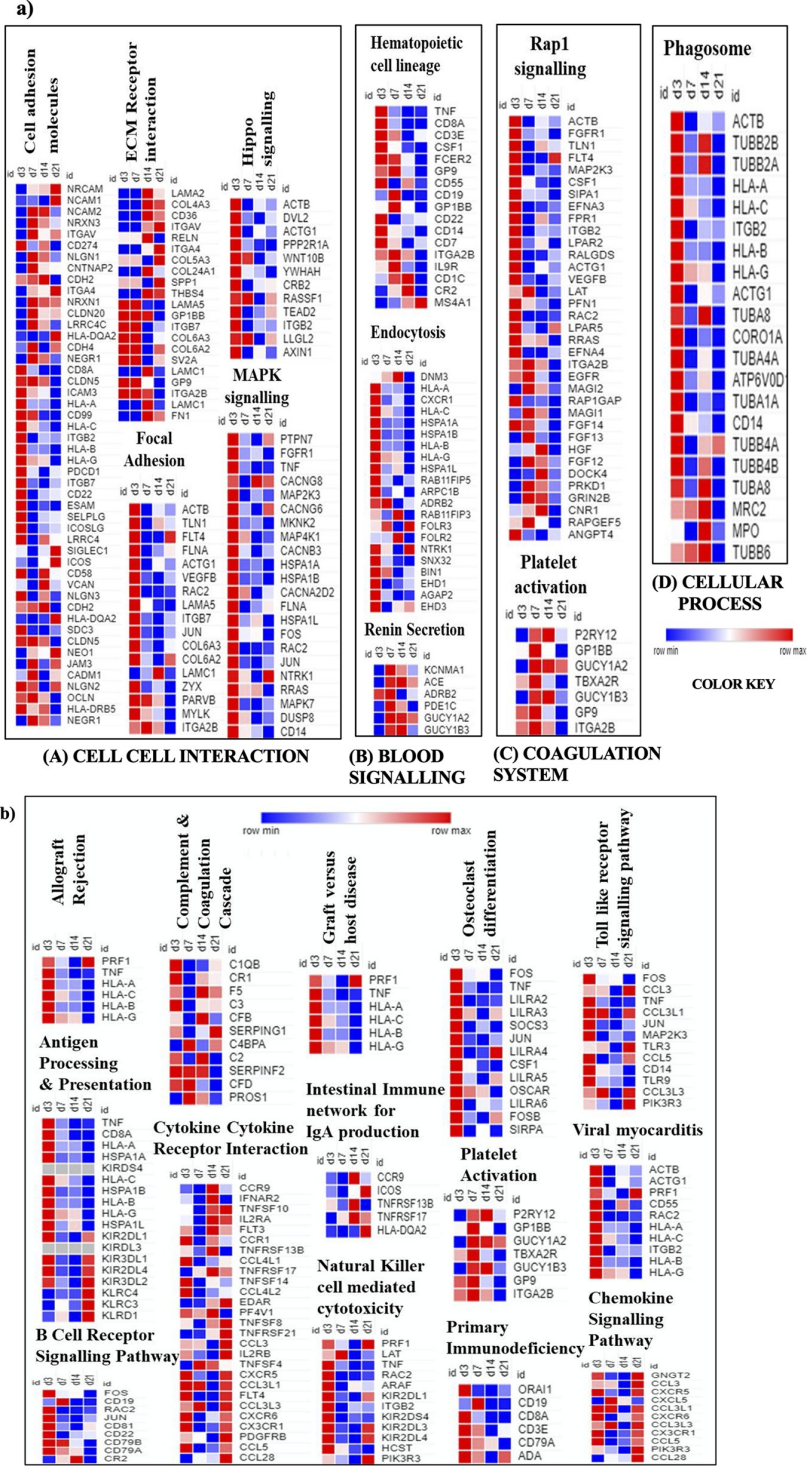
On the basis of the biological functions of gene ontology annotations, **a**. cellular related a) Cell-cell interaction, b) blood signalling c) coagulation system d) cellular process and **b**. Immune system related differentially expressed genes were clustered. The heat maps for mRNA expression at different temporals were clustered and color coded according to the log2 fold changes for each time point versus basal. Expression values of specific genes are represented by colour intensities as shown in the reference colour key.

**Fig 5:**
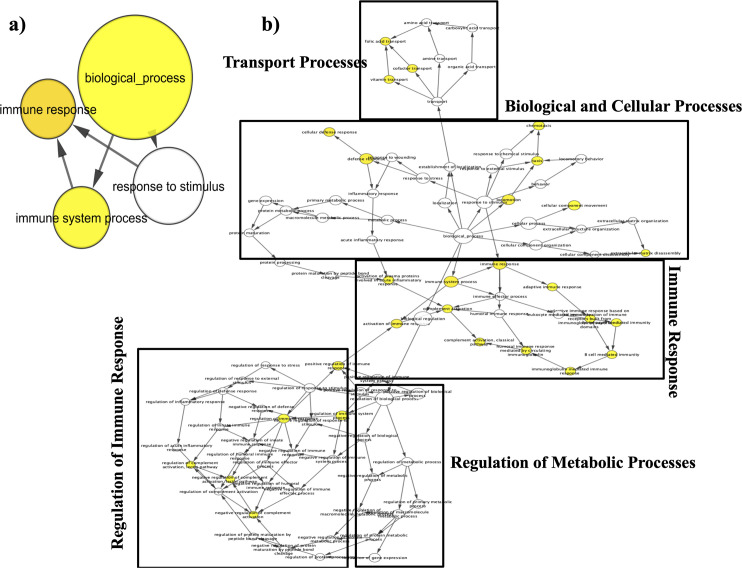
Significant overrepresentation of biological processes **A**) 394 common genes acquired from all different time points analysis. B) 21 common genes obtained from Morpheus gene annotation clustering and 394 genes based on GO terms with differentially expressed genes identified using a log2FC≥ ±0.58 and p value threshold adjusted for less than 0.05 derived using the hypergeometric distribution test corresponding to differentially expressed genes were determined with Benjamini-Hochberg FDR correction with node size representing the number of targets in the GO category. The enrichment GO network was constructed using Cytoscape 3.2.1 with BinGO plug-in. Gene ontologies obtained from different data mining strategies were similar in nature.

After data mining, we sought to understand how expression pattern of these genes occur during different time points post high altitude exposure. Grouping of pathways helped us unify genes in a common category and further comprehend our data. After data compilation broad categories obtained were cell-cell interaction, blood signalling, coagulation system, cellular processes and inflammatory responsive pathways. Separate heat maps were prepared consistent with the gene expression of KEGG pathway enrichment analysis and were further compiled. These KEGG pathways comprised of both down and upregulated expression of different high altitude exposure temporals. First heat map (Fig 4A) chiefly involve four groupings, 1) cell-cell interaction involves: a) cell adhesion molecules (hsa:04514), b) ECM receptor interaction (hsa: 04512), c) focal adhesion (hsa:04510), d) hippo signalling pathway (hsa: 04390), e) MAPK signalling pathway (hsa: 04010); 2) blood signalling involves: a) hematopoietic cell lineage (hsa: 04640), b) endocytosis (hsa: 04144), c) renin secretion (hsa: 04924); 3) coagulation system: a) Rap`1 signalling (hsa:04015), b) Platelet activation (hsa: 04611); 4) cellular processes: Phagosome (hsa: 04144). Second heat map (Fig 4B) largely involved in inflammatory responsive pathways. Pathways shown in heat map were indicative of upregulation of majority of genes at HAD3. Such a time progression hypoxic exposure clustering analysis from genes to biological roles direct us to a preliminary conclusion that maximum genes were upregulated at initial time point ‘HAD3’, some genes were perturbed on subsequent time point, while majority of genes showed a stable response at later time points. Heat map indicated distinct expression pattern and importance of cellular responses at early time point. Interestingly we observed common biological process emerging in independent data mining strategies, suggesting specificity of analysis and also unique nature of gene ontology functions for Kyrgyz. 394 common genes were sorted for overrepresentation of GO terms using cytoscape 3.2.1 with BinGO plugin in FDR correction using Benjamini- Hochberg generated enriched clusters. Enriched terms included Biological process, immune response and immune system process (Fig 5A). Common genes sorted from biological process data mining of 394 genes and from Morpheus gene annotation clustering were *CD274*, *CDH2*, *ESAM*, *LAMC1*, *FOLR3*, *FOLR2*, *GUCY1A2*, *TUBB4A*, *RPS26*, *RPS15A*, *CD19*, *SERPING1*, *C4BPA*, *CCR9*, *TNFRSF17*, *CCL3*, *CCR9*, *CXCL5*, *IL9R*, *PFN1*, *ACTB*. These gene transcripts mined with BinGO plugin revealed biological terms which were clustered further as biological and cellular processes, transport process, immune response, regulation of immune response and regulation of metabolic process (Fig 5B).

### Validation of RNA sequencing experiments using qRT-PCR

**Fig 6:**
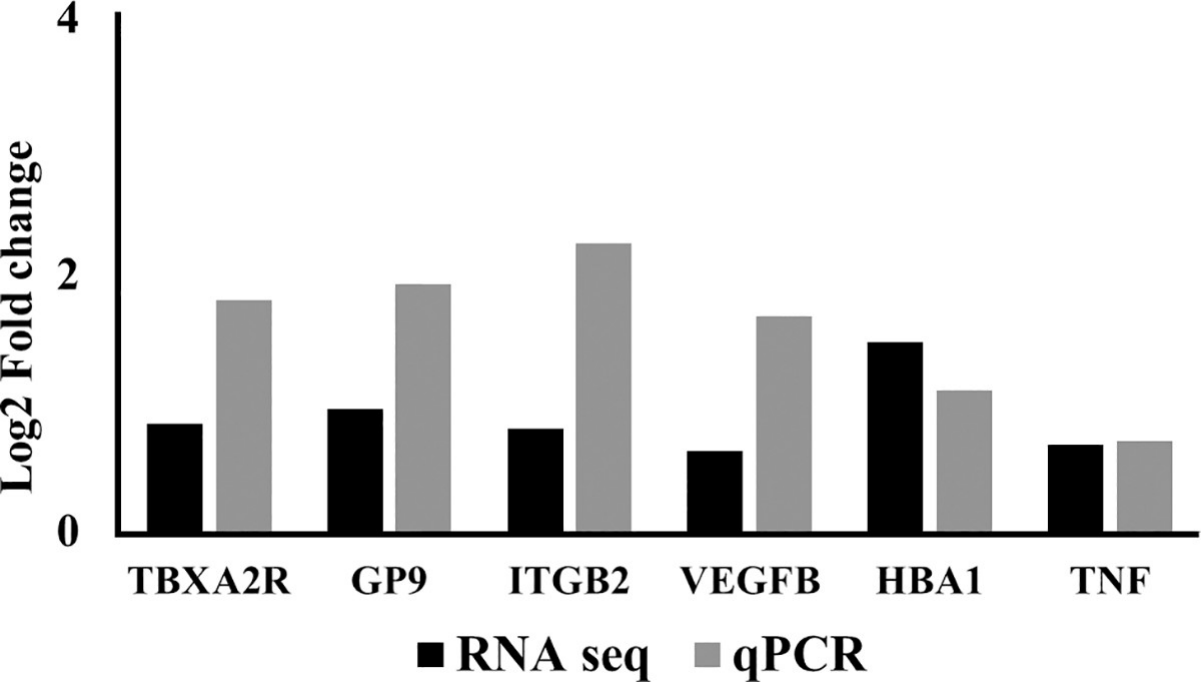
Technical validation of differentially expressed genes using reverse transcription polymerase chain reaction (RT-PCR) showing comparison of relative expression (log 2 values) of selected genes obtained by RNA sequencing and real time PCR experiments. Random selection of six genes was made to validate their expression pattern.

We also performed technical validation of DEG’s using qRT-PCR by selecting random genes which show critical and overlapping involvements in different gene ontologies. Thus, 6 genes were selected. Substantial agreement was observed between the RNA sequencing and qRT-PCR results in all 6 genes. The fold change obtained in each gene from both the techniques showed similar drift (Fig 6). Candidate gene expression was normalized each time in different time points with 18s as the endogenous control.

### Differentially expressed genes network analysis and hub genes identification

GeneMANIA coexpression network of high altitude day3 time point genes was constructed using GeneMANIA cytoscape plugin. After importing the data from cytoscape and running the network analysis, the top 10 genes were evaluated by the 5 topological feature average shortest path length, degree, clustering coefficient, closeness centrality and betweenness centrality. The network obtained from network analysis is provided in S1 Fig. As the network was very complex so we figured out important nodes/genes of the network on the basis of topological features. The most significant genes were different in different topological categories but out of them 3 categories showed overlapping genes (Table 4). Thus we short down the network analysis categories to 3. These 3 categories are degree, closeness centrality and betweenness centrality. The top10 genes involved in degree are *VIM*, *KCNH3*, *CORO1A*, *LY86*, *CD37*, *SH3BGRL3*, *STMN1*, *RHOC*, *IL2RB* and *GRIN2B*. Top 10 genes involved in Betweenness centrality are *KCNH3*, *VIM*, *PDE7B*, *NELL1*, *CACNA2D1*, *GAMT*, *STMN1*, *UNC13C*, *RHOC* and *CENPI*. Top 10 genes involved in closeness centrality are *ZFP641*, *PRSS53*, *VIM*, *KCNH3*, *CACNA2D1*, *SLC29A1*, *NRP1*, *CD37*, *CORO1A* and *TAGLN*. These genes have been mentioned in order of highest to lowest in degree, betweenness centrality and closeness centrality respectively. Vimentin (*VIM*) reflected as the gene with highest degree can be claimed as the hub gene at HAD3 time point. *VIM* also appeared in betweenness and closeness centrality. Other important genes involved in cell-cell interaction and cellular dynamics are *CORO1A*, *CD37*, *STMN1*, *RHOC*, *PDE7B*, *NELL1*, *NRP1* and *TAGLN*.

**Table 4:** Top 10 genes obtained from network analysis of HAD3 DEG’s using three different topological features: Degree; closeness centrality and betweenness centrality. Three categories designate *Vimentin* as the mutual gene.

Gene Name	Degree	Gene Name	Closeness centrality	Gene Name	Betweenness centrality
***Vim***	147	*Zfp641*	1	*Kcnh3*	0.020402
*Kcnh3*	108	*Prss53*	1	***Vim***	0.018953
*Coro1a*	101	***Vim***	0.48668	*Pde7b*	0.011939
*Ly86*	99	*Kcnh3*	0.465002	*Nell1*	0.011187
*Cd37*	99	*Cacna2d1*	0.464321	*Cacna2d1*	0.010118
*Sh3bgrl3*	91	*Slc29a1*	0.461838	*Gamt*	0.009723
*Stmn1*	91	*Nrp1*	0.461165	*Stmn1*	0.009025
*Rhoc*	89	*Cd37*	0.460048	*Unc13c*	0.009023
*Il2rb*	87	*Coro1a*	0.459159	*Rhoc*	0.008951
*Grin2b*	86	*Tagln*	0.459159	*Cenpi*	0.008923

#### Biological validation of physiological, DAVID and GeneMANIA results

**Fig 7:**
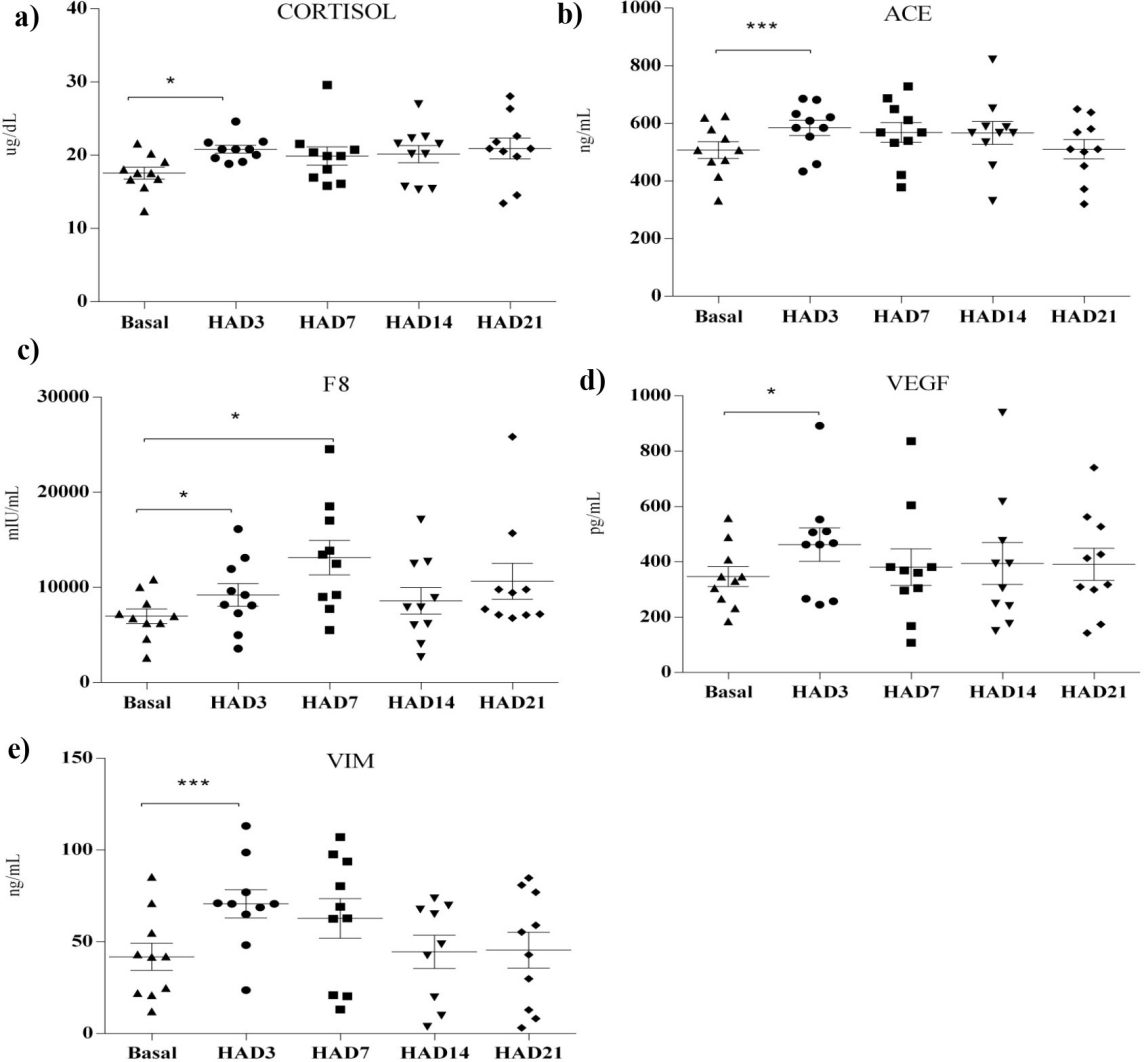
(**a-d**) Dot plots showing biological validation for important markers of the physiological, DAVID and GeneMANIA results obtained at different temporal (**e**) Dot plot showing VIM protein expression at different high altitude time points. Statistical significance is represented as *p<0.05, **p<0.01, and ***p<0.001 at different high altitude time points in comparison to basal.

Proteins were picked to deeply understand the responses acquired by sequential analysis performed. Cortisol expression was measured to confirm the physiological changes. Cortisol increase was ~19% at high altitude day 3 as compared to basal. Further, angiotensin converting enzyme, coagulation factor 8 and vascular endothelial growth factor were picked for validating the pathway enrichment analysis expression levels, ~12%, 32% and 33% increase was observed at D3 time point in comparison to basal respectively. Also, the protein expression level of hub gene obtained from GeneMANIA cytoscape analysis *Vimentin* was observed to be ~69% increase at day 3 time point in Kyrgyz individuals when compared to basal (Fig 7A–7E).

## Discussion

High altitude imposes phenomenon which lead to preliminary pathophysiology and acclimatization benchmarks to survive the same. In our study we found that response to high altitude is complex and depends on time of exposure. Transcriptome analysis using RNA sequencing can provide novel biological insights into the pathways that are critical for high altitude survival. Our study adopted an approach in Kyrgyzstan group to understand the biological basis of high altitude induced acclimatization response in a temporal manner. We used approach in which whole blood gene expression profile at high altitude and control i.e. basal were compared using different bioinformatics tool to find out the novel biological players pertinent to high altitude.

Our study reflected increased systolic and diastolic blood pressure during initial time point and significant increased levels of cortisol in Kyrgyz significantly at D3 (p<0.05) point out towards stress condition after high altitude exposure. Mean pulmonary artery pressure increased considerably on day3 of high altitude made them prone to pulmonary vasoconstriction and increased pulmonary arterial vascular pressure. SpO_2_ has been suggested as a useful tool for prediction of hypoxia. SpO_2_ significantly (p<0.001) dropped at altitude during initial time point HAD3 and regained at later time points. This result is consistent with the previous studies in which lower arterial oxygen saturation has been observed in hypobaric hypoxia [20, 21]. The reason may be increased dead space ventilation, triggered by lower tidal volume and higher breathing frequency [20, 22]. Heart rate significantly (p<0.001) increased on Day7 during initial time point as the blood pressure and mPAP increased contrary with the Tibetans that mPAP did not change and they develop only minimal hypoxic pulmonary hypertension [23]. BP, HR and SpO_2_ helped us monitor the progression of high altitude induced hypoxia and gave a lead for gene expression level changes. Overall, temporal analysis of Kyrgyz physiology helped understand the pattern of responses and cover all phases in this group to study the dynamic changes trend. Physiology parameters reveal time point D3 to be perturbed significantly, thus this phase could be point of analysis. We found a time window where physiological changes are observed and thereafter it subsides gradually. It has given us an opportunity to understand the molecular basis of this phenomenon. In our study when we subjected gene expression data to unsupervised learning we found a similar trend that day 3 has a unique molecular signature compared to other time point.

Further analysing day 3 time point touched upon pathways related to Cellular dynamics and cell adhesion molecules; Rap1 signalling, MAPK, vascular morphogenesis and angiogenesis, Platelet Activation and Hypercoagulation, Angiotensin converting enzyme, hypoxic pulmonary vasoconstriction and immune response which are further separately discussed.

The mechanical interaction between the cell and extracellular matrix influence the cell behaviour and function. Cell adhesion is generally the ability of a single cell to stick to another cell or an extracellular matrix forming an endothelium which is a dynamic organ to regulate vascular tone [24, 25]. Vascular cell adhesion molecules are expressed on activated endothelium. Along with it cell-cell adhesion CAM’s are involved in the transmission of signals across cell membrane [25]. It is reported that continuous hypobaric hypoxia cause altered expression of junctional protein complex of vascular endothelial cells [26, 27]. As evident from our data gene transcripts like integrin *ITGAV*, *ITGB2*, *ITGB7*, *ITGA4 and ITGA2;* in cell adhesion molecule, focal adhesion and extracellular matrix receptor interaction, involvement of collagens *COL6A3*, *COL6A2*, claudins *CLDN5* and other cell adhesion molecules like, *ICAM3*, *CADM1*, *CDH2*, *CD58*, *ACTB*, *TLNN1*, *FLNA*, *ACTG1*, *LAMA5*, *PARVB*, *MYLK* all are upregulated during initial time point in Kyrgyz suggesting modulation of cytoskeleton dynamics as a part of acclimatization to overcome hypobaric hypoxia. Similarly, in Tibetans and Sherpas gene network analysis suggest involvement of collagen and integrin in multiple pathways which are involved in the cellular functions related to angiogenesis [28].

Temporal cellular level changes at high altitude reveal occurrence of endothelial dysfunction. These cell adhesion dynamics might point towards enhanced Rap1 signalling which is evident in our present transcriptome data. Importance of cell dynamics is in several processes like coagulation, angiogenesis etc. Angiogenesis is a process of formation of new blood vessels from the existing vasculature [29]. This process needs cellular level proliferation which is evident by the up regulation of Mitogen activated protein kinase signalling during hypoxia. Evidence suggested role of hypoxia in phagosome formation, as evident in our study, for initiation of phagocytosis by activation of intracellular pathways i.e. MAPK pathway that could mediate the early events required for internalization/phagocytosis [30]. Gene transcripts like MAP2K3 log2Fold change = 0.72, MAPK7 log2Fold change = 0.65, RAC2 log2 Fold change = 0.70 (a small GTPase that regulate cellular responses) [31] and JUN log2 Fold change = 0.71 which is involved in proliferation and survival [32] were upregulated. Rap1 is a Ras family of GTPase that is activated by guanine nucleotide exchange factors (GEFs) and is subsequently converted back to its inactive GDP-bound state by GTPase-activating proteins (GAPs). Ras family of GTPase i.e. Rap1 play a key role in regulation of angiogenesis by modulating endothelial cell functions [33]. Rap1 promotes angiogenic signalling by several factors including growth factors like EGFR, FGF and VEGF. In our study the gene transcript level of VEGFB log2 Fold Change = 0.66, FLT4 log2 fold change = 0.631 and FGFR1 log2 fold change = 1.412 has been noticed to be upregulated on Day3 time point thereafter the expression got down regulated. VEGF increase vascular permeability and allow extravasation of plasma proteins thus forming a primitive scaffold for endothelial cell migration. VEGF protein in Kyrgyz show significant (p<0.05) upregulation at HAD3 supports and validate gene data. Angiopoietins, proteins that play important role in vascular development and angiogenesis that is important in hypoxic stress [34]. In our present study ANGPT4 is upregulated at day3 time point and thereafter it downregulated. ANGPT’s mechanism by which they promote angiogenesis is involved in regulation of endothelial cell interaction with other vascular cells. FGFR2, NF1 and RasGef4, are the candidate genes which function in the Ras/ERK signalling pathway and which commonly promotes angiogenesis with the HIF pathway under hypoxia [35]. In our study gene transcripts like RAPGEF5, RAP1GAP played an important role in activating the RAP1 signalling at initial time point of altitude.

Arterial venous thrombosis is observed under some cases of acute hypobaric hypoxia wherein activation of platelets has been observed during hypoxia exposure in human and animal studies [36–38]. Identification of membrane glycoproteins like GP9, GP6, GP4 and integrin subunits like ITGA2B, ITGB3 has been reported in study in which volunteers exposed to continuous hypobaric hypoxia. Activation of membrane glycoproteins and integrin involvement has important role in platelet signalling and thrombosis [39, 40]. In our study, activation was reflected during day7 of temporal analysis. Activation of membrane glycoproteins gene transcript GP1BB log2fold change = 1.78 is observed on Day7 of high altitude. GP9 log 2 fold change = 0.958 gene transcript is observed to be slightly upregulated during Day3 but major upregulation is observed at day7 time point. Integrin activation ITGA2B log2 fold change = 0.69 and 0.81 was observed at time point day3 and day7 respectively. This might indicate platelet activation not immediately after exposure to altitude rather at later time point. TXA2 is responsible for multiple biological processes through the cell surface TXA2 receptor, or T-prostanoid (TP) receptor [41, 42]. Activation of gene transcript TBXA2R log2 fold change = 1.07 is also observed at HAD7 in Kyrgyz. This gene transcript is involved in the platelet aggregation. During hypoxic microenvironment TBXA2R act as paracrine mediator due to its abundant expression in platelets surface [42]. TXA2 is responsible for multiple biological processes through its cell surface receptor TP. High altitude has been associated with hyper coagulation state due to various environmental stresses and also RAP1 activation, as discussed in last section, in the Kyrgyz might be associated with platelet activation. This is confirmed by the studies which indicate that about 8% of the known proteins expressed in platelets are small GTPase [43, 44]. RAP is the most abundant of protein in platelets [45]. Rap1 activation is evident in our transcriptome data regulates multiple responses in platelets, most notably integrin and glycoprotein activation on high altitude day7 in Kyrgyz. This is also supported by the other studies that rap1 activation leads to platelet activation [46, 47]. Also, activation of platelet proteome/ reactivity correlates with a prothrombotic phenotype [38]. We measured coagulation factor 8 (F8) in serum and found to be upregulated during HAD3 which supports activated platelet gene transcripts. Platelet activation also induces Rap1 activation. It is a kind of cycle in which both promote each other. Platelet stimulation involves the interaction of ADP with platelet receptor P2Y12 leading to inhibition of RASA3 which in turn prevent the conversion of RAP1-GDP. Thus RAP1 remain in activated form and result in fast and sustained integrin αIIβ3 activation leading to coagulation effect [45].

Angiotensin converting enzyme is a protease which is capable of cleaving two amino acids from angiotensin 1 and form Angiotensin II, which is a powerful vasoconstrictor. *ACE* is found highest in pulmonary circulation and form angiotensin there causing hypoxic pulmonary vasoconstriction in humans [48, 49]. This alteration in systemic blood pressure is due to vasoconstriction. *ACE* log2 fold change = 0.6 gene transcript in our study is upregulated in the Kyrgyz on day7 of high altitude. Previous studies on Kyrgyz individuals revealed the polymorphism associated with *ACE* gene transcript. It was studied that I/I genotype was more strongly related with pulmonary hypertension in Kyrgyz than in comparison to other phenotypes like I/D, D/D [12]. It has been studied that during initial 2–3 days results in very high level of renin due to strong sympathetic stimulation of kidney as per effects of hypoxia [50]. These very high renin level increases the angiotensin I levels and further angiotensin II. ACE levels in our study show significant (p<0.001) increase in Kyrgyz at HAD3 as compared to basal time point. This is suggestive of initial vasoconstriction and increased blood pressure and mPAP as evident from our physiology analysis. In present study physiological data mPAP and BP remained elevated even at later time points of altitude exposure.

In present study, it is noticeable that immune system was highly regulated during temporal analysis in Kyrgyz individuals. During HAD3, which is the initial time point for estimating the gene level changes in this group, it is evident that antigen processing and presentation, B cell receptor signaling, Natural Killer cell mediated cytotoxicity, Toll like receptor signaling pathways all are up regulated. The gene transcripts involved were different Major histocompatibility complexes, *HLA-A*, *HLA-B*, *HLA-C*, *HLA-G* log2 fold change = 0.65, 0.82, 0.91, 0.64 respectively are found on the surface of antigen presenting cells (called as APCs) for presenting peptides/antigens at cell surface; gene transcripts like *CD22 CD79A*, *CD81* log2 fold change = 0.80, 0.92, 0.81 respectively which mediate B-cell B-cell interaction; gene transcript like *LAT*, *ARAF*, *PRF1* log 2 fold change = 0.62, 0.65, 0.81 respectively all induce T cell activation. MHC activation during initial time point is a protective mechanism of Kyrgyz at high altitude for presenting antigens and reflects boosted adaptive immunity. Similarly, upstream regulated pathway is Complement and coagulation cascade. Activation of gene transcripts like *C2*, *C3*, *C4BPA*, *CFB*, *C1QB* during day7 also supports the activation of immune system for fighting against antigens. Along with adaptive immunity, innate immune system is also getting upregulated during initial temporal analysis in Kyrgyz individuals. Further, during Day7 of time point analysis cytokine-cytokine receptor interaction is getting down regulated. Cytokines, chemokines and their receptors like CCR9 log 2 fold change = -1.691, TNFSF10 log2 fold change = -0.583, CCL4L1 log2 fold change = -0.99 were down regulated which is indicative of reduced migration, activation of immune cells and future pathogenesis of human disease as the individuals getting acclimatized. Chemokines are essential players in immune and inflammatory reactions as well as infections [51, 52] and this reveals a possible protective mechanism against inflammation or infection. Investigation has been done on probable association of inflammation in the pathogenesis of acute mountain sickness [53]. It has also been reported that epithelial gene transcript perturbation during hypoxia causes inflammation [54]. As, epithelial cells provide mucosal barrier thus cellular dysfunction cause less defense of mucosal barrier function. It is evident from our gene data that the epithelial function is disturbed during hypoxia exposure and this might also be a cause of strong inflammatory upregulation during initial time point. Endothelial activation and inflammation has strong correlation [55].

It is evident from the clustering of gene ontologies analysis that initial time point HAD3 is very important in terms of dynamic changes occurring in Kyrgyz. Thus, we further analysed this time point to find out the involvement of important genes and their associated functions. Genes obtained from the degree; betweenness centrality and closeness centrality were checked for their specific roles. It was found that among the genes 50% (i.e. 5 genes out of 10 in each in each category is involved in cellular level changes and cell to cell interaction. *VIM*, *CORO1A*, *CD37*, *STMN1*, *RHOC*, *PDE7B*, *NELL1*, *NRP1* and *TAGLN*. Involvement of all these genes during hypoxia at cellular level suggests hypoxia affect structure and function of endothelial cells. Among it, *VIM* is lying in all 3 analysis and has the highest degree and could be pointed as a hub gene and rest all genes lie in at least 2 of the category. Hypoxia exerts effect on *Vimentin*, an important component of endothelial intermediate filament network, which help maintain structure and function of endothelial cells. As we have observed from the KEGG pathway analysis that activation of signalling pathway like MAP Kinase, cell adhesion molecules, focal adhesion and extracellular matrix receptor interaction pathways can alter the actin cytoskeleton and alter the endothelial cell migration, and barrier permeability during hypoxia. Thus, in the KEGG pathway analysis, the top pathways were associated with the cell-cell interaction which is in consonance with the hub gene functions. It has been shown that hypoxia causes redistribution of *Vimentin* to a more soluble and extensive filamentous network that could play a role in endothelial barrier stabilization. Results of our study support other studies which suggest up regulation of *Vimentin* in hypoxic cells [56, 57]. It has also been observed that in endothelial cell surface *Vimentin* binding peptide induces angiogenesis under hypoxic conditions [58]. In our study *VIM*, on protein level, is significantly up regulated with p≤0.001 at HAD3. In brain capillary endothelial cells upregulation of *Vimentin* has been reported [59]. In bone marrow endothelial cell line, *Vimentin* has been shown to regulate focal adhesion [60]. Other critical genes have been reported to be involved in cellular dynamics during hypoxic acclimatization.

Being multifactorial in nature, high altitude is characterized with changes in physiology, vascular tone, inflammatory milieu, thrombotic factors and cellular dynamics. At molecular level high altitude is associated with an extensive alteration of systemic gene expression. Such extensive changes at high altitude induce pathophysiology which is not caused due to perturbation of single pathway but rather a cumulative alteration of many signalling modules. In summary, by using a series of analysis, genomics and bioinformatics we illustrated the decisive time frame i.e. high altitude day 3 for maximum perturbations in genome of Kyrgyz individuals cross validated with physiological and biochemical markers. Temporal analysis suggested cell-cell interaction and immune responsive genes to be majorly involved in the early phase of acclimatization response. Endothelial activation (cell-cell interaction) and immune response generation/ inflammatory response are both interrelated. It is evident from the clustering of gene ontologies analysis that initial time point HAD3 is very important in terms of dynamic changes occurring in Kyrgyz. Moreover, in this study we also tried to find the nodal point of the gene interactive network and candidate gene controlling many cellular interactive pathways VIM, CORO1A, CD37, STMN1, RHOC, PDE7B, NELL1, NRP1 and TAGLN and the most significant among them i.e. VIM gene was identified. These hub genes may be used in the future as a biomarker and therapeutic target for accurate diagnosis and treatment of high altitude induced hypoxia. This study will contribute to the knowledge of the molecular mechanisms underlying the acclimatization of Kyrgyz towards high-altitude environment.

## Limitations and future prospect

We acknowledge that study was limited to 21 days at high altitude and was not investigated after descent from high altitude to baseline. Further similar studies can be conducted at similar altitude on larger number of volunteers for assessing the effect after 21 days and after descent to baseline.

## Supporting information

S1 File Input file for analysis with gene name and respective log2 fold change values at high altitude D3, D7, D14 and D21 as compared to basal. (XLSX)

S1 Fig The pathway network obtained using GeneMANIA coexpression network analysis. (TIF)
