# Supplementary figures and images for: Temporal transcriptome analysis suggest modulation of multiple pathways and gene network involved in cell-cell interaction during early phase of high altitude exposure
Source: PLoS One. 2020 Sep 10;15(9):e0238117. doi: 10.1371/journal.pone.0238117 (PMC7482924; doi:10.1371/journal.pone.0238117)

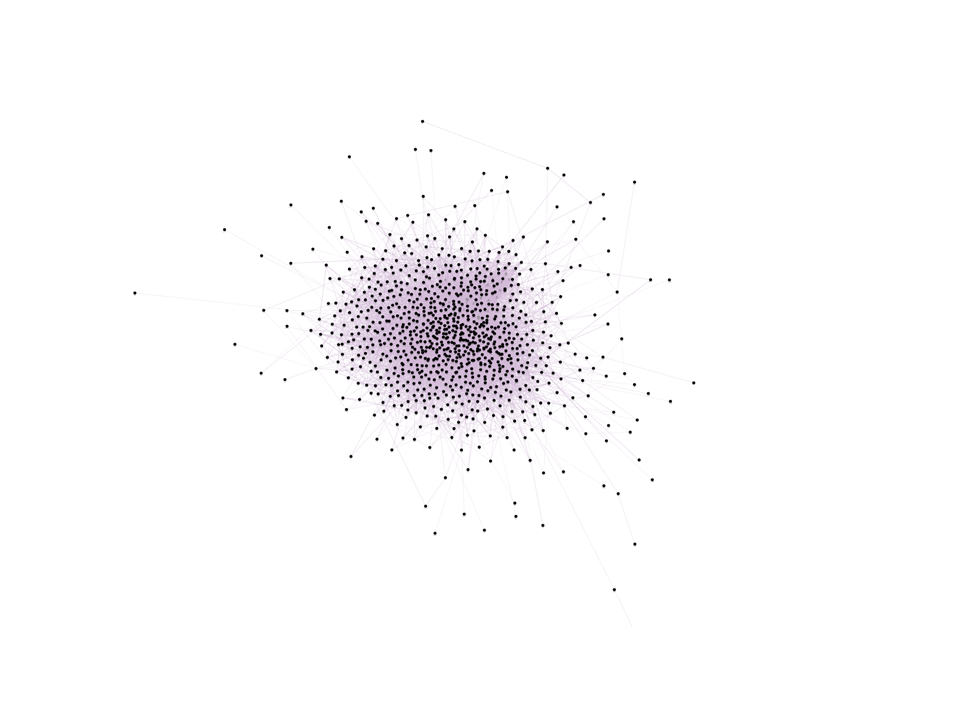

Supplement: S1 Fig — (TIF) [file pone.0238117.s002.TIF]
